# Mine Water for the Generation and Storage of Renewable Energy: A Hybrid Hydro–Wind System

**DOI:** 10.3390/ijerph18136758

**Published:** 2021-06-23

**Authors:** Héctor Álvarez, Guillermo Domínguez, Almudena Ordóñez, Javier Menéndez, Rodrigo Álvarez, Jorge Loredo

**Affiliations:** 1 Department of Mining Exploitation and Prospecting, School of Mining, Energy and Materials Engineering, University of Oviedo, 33004 Oviedo, Spain; UO259322@uniovi.es (H.Á.); alvarezrodrigo@uniovi.es (R.Á.); jloredo@uniovi.es (J.L.); 2 SADIM Engineering, 33900 Ciano, Spain; UO258347@uniovi.es (G.D.); javier.menendez@sadim.es (J.M.)

**Keywords:** mine water, underground mining reservoir, energy storage, underground pumped hydroelectric energy storage

## Abstract

Mine water is normally considered as waste that has to be managed. However, new applications are increasingly being sought for the water that floods mining voids, especially in relation to its use as an energy resource. The worldwide energy market, within the current transition framework, is searching for creative approaches to produce and store clean energy. In particular, underground pumped hydroelectric energy storage systems (UPHS) constitute efficient and flexible alternatives to deal with intermittent renewable energy sources. In this work, a UPHS is designed using the mine water and the voids of a closed coal mine in Asturias (North-west Spain) as a lower reservoir. Moreover, this system is combined with a wind energy generation facility and the efficiency of the hybrid system is evaluated. With an investment cost of EUR 193 M, a 40 MW UPHES joined to a 60 MW wind farm would generate benefits of about EUR 54 M in 40 years. The reduction in CO_2_ emissions (29,000 equivalent tons per year) and the social benefits in a traditional mining area are other intangible advantages of this system.

## 1. Introduction

Since the Industrial Revolution, fossil fuels have undoubtedly been the dominant energy sources, both either in electricity generation or in transport and heating. However, in recent years, renewable energy sources have gained importance in the field of electricity generation. Among the reasons for this turnaround, we can find different factors. First, the fulfilment of the guidelines set in the Paris Agreement of 2015, which established the roadmap to reach the zero-carbon emissions goal. Moreover, a significant evolution of renewable generation technologies, mainly wind and solar energy, has enabled a reduction in their generation costs. So much so that, currently, more than two thirds of the world’s population live in countries where solar or wind energy, if not both, are the most economical sources of electricity generation [1]. On the other hand, it is estimated that world energy demand will increase by 62% before 2050 [2]. Thus, it is expected that a large part of the growth in installed power throughout the world will be linked to the rise of renewables, which will become the main source of energy, first in Europe, and then in the rest of the world. Nevertheless, this will not be possible unless there is a profound transformation of the electrical system. Energy storage installations in particular can guarantee a continuity of supply that renewable energies are unable to provide, providing a solution for grid stability in the integration of non-manageable renewable energy sources [3,4].

This transformation involves developing a profitable and reliable storage technology that makes it possible to increase the availability of this remarkable energy, alleviate congestion in the electricity grid, and mitigate power variations through the ancillary services. Regarding costs, although storage has been and continues to be an expensive technology (an average of USD 187 MWh^−1^), it is expected that by 2040 the levelized cost of energy storage will decline by approximately 64% from current values to USD 67 MWh^−1^ [2]. Currently, conventional generation systems such as combined cycles or thermal plants are the most reliable back-up technologies for large-scale power systems. Nevertheless, storage technologies will play a very important role in the near future, thus allowing renewable energies to become the leading source within the electricity sector [5].

According to Newman and D’Aprile [6], energy storage consists mainly of absorbing energy to release it later so that it can be generated at one time and used at a more convenient time. This storage can take place in various ways: mechanical (compressed air energy storage (CAES)), pumped hydroelectric storage (PHS) and underground pumped hydroelectric storage (UPHS) systems, electrical and electromagnetical (supercapacitors), thermal (heat recovery), and chemical and electrochemical (hydrogen, fuel cells, batteries). This work focuses on the underground pumped hydroelectric energy storage (UPHS) systems inside underground mines. These systems take advantage of the mine water, which can be used to generate energy in closed, flooded mines, storing the surplus energy generated by renewable sources. In particular, a UPHS–wind hybrid system is described. The paper aims to analyze how abandoned mines can be economically reused for the sustainable implementation of energy generation and storage.

## 2. Underground Pumped Hydroelectric Energy Storage (UPHS)

Although the concept dates back to the early 20th century [7], the interest in this technology has increased in recent years. Thanks to these systems, the management of energy produced by renewable sources is optimized, the stability of the grid is guaranteed and the supply to the electrical system is assured [8]. A generic pumped hydroelectric energy storage (PHS) plant is made up of an upper and a lower reservoir connected by a driving line between them and a pump-turbine unit [9]. The potential energy stored in the water of the upper reservoir is used for the generation of electricity according to the system requirements [10]. During off-peak times, the water is pumped from the lower reservoir to the upper one using electrical energy from the grid, and during periods of high demand, the water flows back to the lower reservoir through the turbines to produce electricity. The storage capacity of the plant is given by the height difference between the two reservoirs and the volume of the stored water [11]. Among its most remarkable features, this technology has a short start-up time, technically known as fast ramping, and a cycle efficiency that ranges between 70% and 80%, although values of 87% have been reached [12]. In terms of profits, this type of storage system is based on on the difference of the energy price during the day. When the price is low, the energy is consumed from the grid to pump water from the lower reservoir to the upper one. When the price is high, the water drops from the upper reservoir, driving the turbine to produce electricity, which is fed back to the grid. In other words, energy is sold on the market at a higher price than it is bought. Assuming an efficiency of 70%, the price for energy sales should be at least 1.4 times higher than the price during the hours of pumping for the system to be economically viable [13].

In UPHS plants in particular, the lower reservoir is located underground. The design of the UPHS plants came from the necessity to expand conventional pumping stations, which are limited by two decisive factors: (i) topographic restrictions, since a competitive hydraulic head between both reservoirs is needed; and (ii) public acceptance mainly related to the environmental impact and the land use when constructing these plants on the surface. UPHS plants allow massive amounts of energy to be stored, which avoids these problems, and they also achieve a high operating efficiency, a large storage capacity (several hours of supply), and a long, useful lifespan [14]. In UPHS plants, the lower reservoir can be built directly under the upper one, thus reducing the horizontal distance between the two reservoirs (and the length of the piping) and maximizing the hydraulic head of the installation. For this reason, a UPHS plant can produce more energy than a conventional pumping station that has the same reservoir dimensions [15].

The installed power (W) of a UPHS plant is given by the following general equation:

P = ρ · g · Q · H · η(1)

where ρ is the water density (1000 kg·m^−3^), g is the gravitational acceleration (9.81 m·s^−2^), Q is the water flow through the pressure pipe (m^3^·s^−1^), H is the net hydraulic head (mH_2_O), and η is the efficiency of the turbine and the generator.

### UPHS Plants in Abandoned Mines

Although the underground reservoir in a UPHS plant can be drilled, common underground or open pit mines are proposed for this purpose, as Harza first used in 1960 [16,17,18]. Hydroelectric energy can be produced and stored using inactive underground mines, so that pumped storage can be established between a reservoir placed on the surface (or at the upper levels of the mine) and a lower deposit communicated by a driving line equipped with turbines. Although major components can be located underground, an outer upper reservoir will reduce costs. The underground reservoirs can be built entirely new or the existing mining voids can be reused. Hence, the construction of UPHS plants takes advantage of abandoned underground mines, presenting a series of additional advantages compared to conventional UPHS plants: (i) The galleries allocated both for the lower reservoir and the electromechanical equipment have already been excavated, reducing considerably the investment costs of the installation. (ii) The access shaft is already built, which allows access to the different underground levels of the UPHS plant. (iii) The mine has its own underground pumping infrastructure (pumps, pumping chambers connected to the network, and a consolidated pipe network). (iv) Both the initial filling and the evaporation replacements of the upper reservoir can be carried out using the mine water flooding the mine, so a river would not have to be diverted to fill it, avoiding negative environmental impacts during the construction and exploitation stages.

There are no commercial scale UPHS plants using mining structures, but new projects and feasibility studies are increasingly being published. That is the case in the reconversion of the closed Prosper-Haniel mine (Bottrop, Germany) to a 200 MW UPHS plant, with an annual water flow of 1 Mm^3^ and a hydraulic head of 1200 m, producing 3 GWh [19]. Estimates suggest a theoretical output of 10 GW and a storage capacity of 40 GWh in suitable German mines [11]. It has also been suggested that the existence of very deep non-flooded galleries, the high underground storage space, and the abundance of water make South African gold mines exceptionally suitable structures for the construction of UPHS plants [20]. There are other examples in Europe and the USA, as well as several techno-economic feasibility studies, including [14,21,22,23] among others. Exhausted, stable, and recently closed mines are preferable for UPHS plants. Mines that could be affected by dissolution processes, such as salt deposits, or those where explosive or toxic substances could be released, should be eluded, as well as those whose infrastructure is preserved as industrial–historical heritage [24].

## 3. Mine Water

When the mining activity intercepts the water level, it is necessary to establish a pumping scheme to dewater the exploitation. Once the mining work is completed and the mine closes, if pumping ceases, the water will progressively flood the mining voids according to a process known as “groundwater rebound” [25]. The flooded voids thus constitute an underground reservoir that will behave like a pseudo-karstic aquifer, in which the porosity is due to (i) the mining galleries and the void left by the extracted ore, (ii) the fractures induced by the mine workings in the overlying massif, and (iii) the natural porosity of the massif itself [26].

During the mine flooding process, the higher the infiltration recharge and the lower the volume of the voids to be filled, the faster the water level will rise [27]. If sulphides are present in the bedrock (e.g., metal, coal, or uranium mining, etc.), they will be oxidized in the mining cavities during the active life of the mine; thus, when they are flooded during the groundwater rebound, the water quality may worsen (acid mine drainage, AMD), causing a negative impact, but this is not always the case [28,29]. Discharges of mine water to the surface will occur when the flood water level reaches the lowest mine adit or any permeable layer hydraulically connected to the mine workings. Since uncontrolled spills are undesirable, pumping is usually restarted and regulated so that the discharge is equivalent to the recharge, in order to maintain a permanent and secure flood level. This artificial underground reservoir can be used in different ways: industrial or drinking water supply, support for ecological river flows, and energy use, such as geothermal and hydroelectric. In that case, a residue (mine water) would be used as a resource, in an efficient implementation of the circular economy concept. The economic use of this water can help to offset the unavoidable costs of pumping and to revitalize former mining areas now in decline.

## 4. Study Area

**Figure 1:**
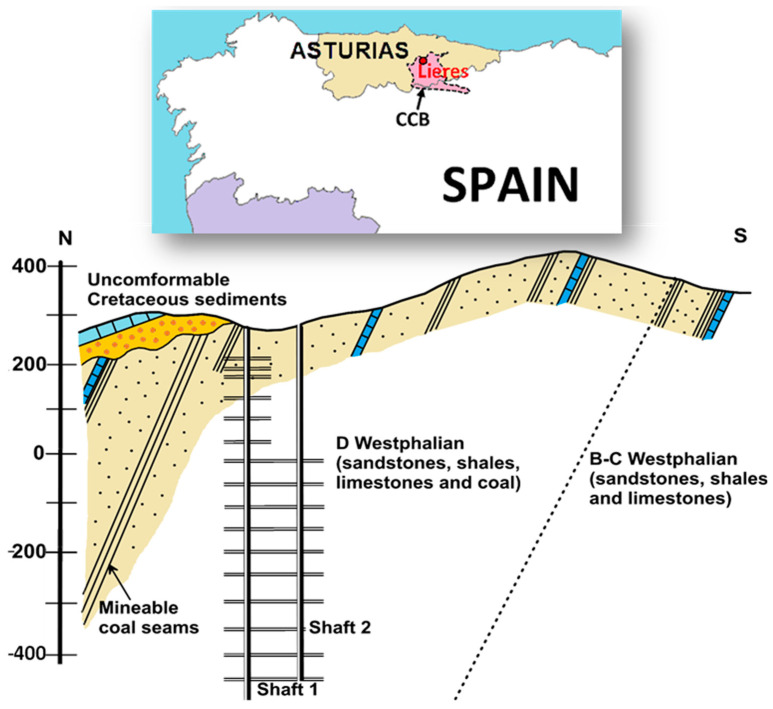
Location of the study area. North–south geological cross section through the Lieres mine.

This study applies to the deepest coal mine (Lieres) in the so-called Asturian Central Coal Basin (CCB) in North-west Spain (Figure 1). The CCB was the main coal-producing area in Spain for 200 years, but the mines have been gradually closing down. In some of these mines there are projects for the geothermal use of mine water [30], among others. The Lieres mine is located in Siero, a village with 12,700 inhabitants.

### 4.1. Geological Context

The CCB is one of the units into which the Cantabrian Zone, in the north of the Iberian Massif, has been classically divided [31]. The CCB comprises a very thick sequence of siliciclastic rocks (paralic), which includes subordinated limestones, from Namurian to Westpahalian D in age. Locally, the coal-bearing succession found in the surroundings of the Lieres mine belongs to the upper series (“Sama Group”) and it is mainly constituted of lutites, but also sandstones (litharenites) and coal seams, as well as Carboniferous limestone thin levels (near the boundary between Westphalian C and D), forming a fan-delta cycle. The coal extracted in the Lieres mines is of the bituminous-C type following the ISO 11760 classification, and the whole series lies within the diagenetic domain, with a vitrinite reflectance of between 0.6% and 1% [32]. The thickness of the coal seams varies between 0.5 m and 2.5 m. Overlying this Carboniferous basement, to the north of the Lieres mine shaft, there is a Cretaceous homoclinal structure, which is constituted by Albian sandstones and microconglomerates, followed by a group of Cenomanian limestones, marls and sandstones (Figure 1).

The tectonic and structural analysis of this area indicates that the contact between the Palaeozoic basement and the Mesozoic cover takes place by means of an angular discordance. Both series show a north dip direction, 70° for the basement and about 30° for the cover. Carboniferous rocks are highly tectonized and form the north limb of a Variscan anticline.

### 4.2. The Lieres Mine

This mine, similar to other mines in the CBB, began its coal production by the end of the 19th century, by means of “mountain mining”: horizontal galleries were drilled from the valley level to the highest coal outcrops, so the extracted coal was evacuated by the bottom gallery. These mine workings were drained by gravity without pumping.

The Solvay company bought the Lieres mine in 1903. The resources extractable by mountain mining were exhausted during the first decade of the 20th century and, since demand for coal increased during the First World War, the first vertical shaft for underground mining was finished in 1916. Activity continued for the next two decades, and a second shaft was drilled in the 1940s. More than 600 miners were working in the mine in the most active period. The first shaft, with 16 levels, is 780 m deep and both shafts (separated by a distance of 50 m) are connected on 10 levels. The mine was integrated in 1995 into the public mining company HUNOSA, which kept it active until its final closure in 2001.

**Figure 2:**
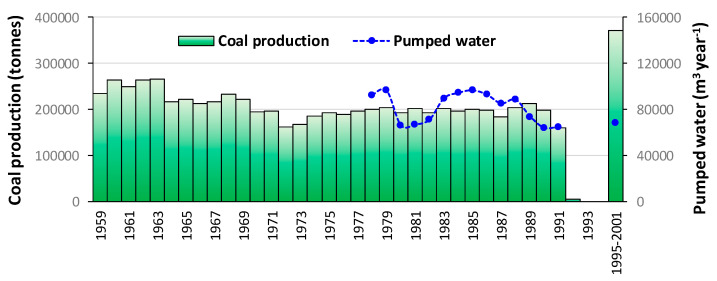
Majority coal production and pumping rate of the Lieres mine.

The coal was extracted by hand, by means of pneumatic hammers in a longwall with the form of inverted stairs, with timber support and backfill, a method used for subvertical (>45°) coal seams. An average of 200,000 tons of coal per year were extracted from the mine (Figure 2) totaling more than 7 million tons (data obtained from HUNOSA and the historical archives). Considering the coal density (1.8 t·m^−3^) and that the residual void is 40% of that occupied originally by the coal, a total volume of 1.6 Mm^3^ was left as empty space. Moreover, according to the HUNOSA records, the galleries in the Lieres mine have a total length of 242 km; assuming a midsection of 10 m^2^ for those galleries, they suppose a void volume of 2.4 Mm^3^. Thus, the total volume of the voids in the mine is more than 7 Mm^3^. This figure was underestimated in previous studies, such as in [13], that did not take into account all the coal mined at the mine. The average pumped flow when the mine was dewatered was around 80,000 m^3^ year^−1^ = 2.5 L·s^−1^ (Figure 2). Step pumping was established to lift the water 780 m; there were three pump rooms in the galleries at depths of 290, 600, and 660 m, with a capacity of 500, 630, and 150 m^3^, respectively. The total pumping power was 1400 kW. This pumping was stopped after the mine closure and now the mine is being flooded.

### 4.3. Hydrogeology

**Figure 3:**
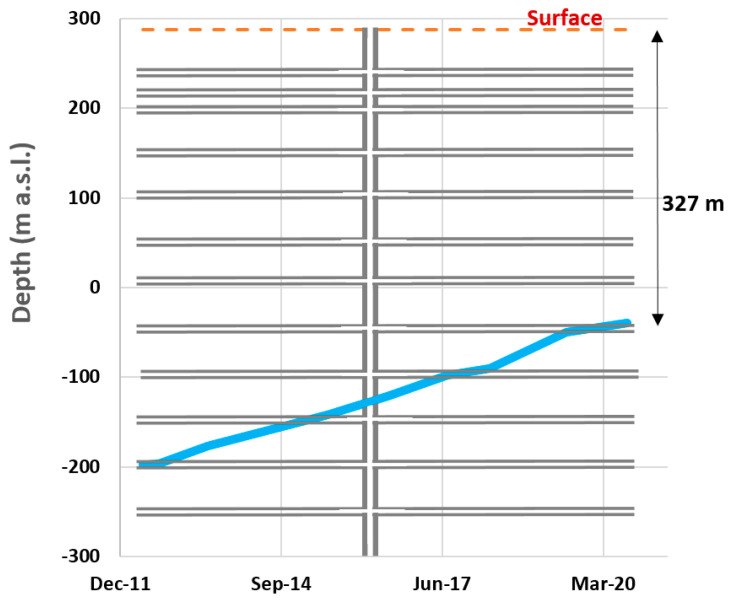
Groundwater rebound (flooding) in the Lieres mine.

From a hydrogeological point of view, CCB Westphalian series have low permeability; no significant aquifers have been defined and the water that the mine receives comes from rainfall infiltration on the related basin. In particular, the Lieres mine is popularly known as a “dry mine” on a regional scale, as the recharge received by infiltration is much lower than in other mines in the CCB. This low recharge is principally due to the absence of mine workings at depths below 60 m from the surface. This prevents mining-induced fractures from reaching the surface and promoting infiltration, unlike other mines in the CCB, which tend to receive more infiltration even from losing streams [26]. Figure 3 shows the water level rise during the flooding of the mine. The mine is currently flooded to a depth of 320 m from the surface and the water level rises very slowly, with an average rate of 19 m per year. The rise in the water level is logically dependent on precipitation; however, water rises more slowly as it reaches the mine levels (greater volume of open galleries to fill) and more quickly between mine levels. Flooding was faster initially, due to a lower volume of mine workings at a greater depth.

**Figure 4:**
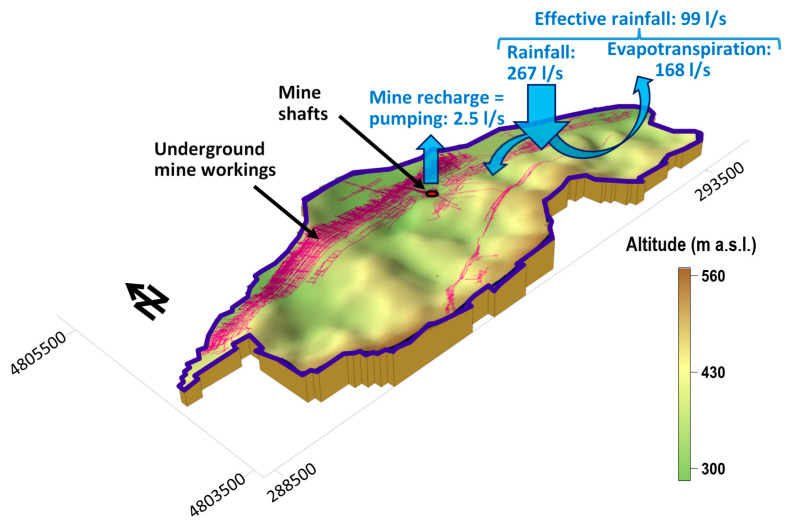
Recharge basin of the mine and conceptual hydrogeological model.

In terms of climate, the average rainfall in Lieres is 950 mm year^−1^, and the average annual temperature is 13.8 °C. The Thornthwaite evapotranspiration is estimated in 63% of the precipitation, so the annual effective rainfall is about 350 mm. The basin that receives the recharge of the Lieres underground reservoir covers 8.8 km^2^ (Figure 4). Supposing that the pumped flow equals the recharge within that basin, only 2.5% of the effective rainfall infiltrates into the mine. The total capacity of the mining reservoir (around 7 Mm^3^) would have to be filled with water to achieve complete flooding, but before that, pumping will probably have to be resumed to keep a safe flood level.

The quality of the water that is currently flooding the mine is not known, but an analysis of the mine water that was pumped when the mine was still active (1998) reveals that the water is not AMD (pH: 8.5; 0.3 mg·L^−1^ Fe; 170 mg·L^−1^ sulphate; 2.0 mS·cm^−1^; 28 mg·L^−1^ suspended solids). Notwithstanding, the quality of the water should be carefully monitored because it can affect the operation equipment, particularly if precipitation of Fe oxy-hydroxides or calcium carbonate occurs, since the UPSH activity might increase the oxygen partial pressure [33,34].

This work involves a UPHS system for the Lieres mine, which was selected for its great depth, for being very close to the potential consumers, and for having little recharge, so that the pumping costs to maintain a certain level of flooding are reduced. The UPHS plant will use the mine to house most of the installation and the lower reservoir, thus greatly reducing the civil engineering costs. The upper reservoir is located at the surface, within the mining site.

## 5. Design of the UPHS Plant

In this case, the existing mine voids will be used to create the lower reservoir, instead of excavating new tunnels, in order to reduce the investment costs. The main components of the plant are described below.

Upper Reservoir: The total volume of this reservoir is 300,000 m^3^, but only 250,000 m^3^ will be available since it cannot be completely filled or emptied for safety reasons.

Pressure pipe: This is the vertical pipe that will run through the interior of the access well (mine shaft) and will be responsible for guiding the water flow from the upper reservoir to the underground plant. In this case, a three-section S355 J2+N type steel pipe with two 90° elbows was selected, with welded joints and thicknesses increasing downwards towards the powerhouse.

Transformers hall: This gallery (25 m long, 6 m wide and 8 m high) will house both the main transformer and the auxiliary services transformer. Sometimes, the transformers hall can be located on the surface, such as in the UPHS plant designed for the Prosper-Haniel mine [35].

Submergence gallery: This is a requirement for the turbines in order to keep a minimum water level above them, i.e., it must be always full of water even when the lower reservoir is at its minimum level of exploitation. In this case, the vertical shaft is 25 m high and 5 m in diameter, providing the turbine with a 25 m submergence level.

Powerhouse: This will be located in a gallery at a depth of 600 m, separated from the transformer room in order not to have all the electrical equipment together with the turbomachines and the motor/generator. Dimensions of 30 m long, 16 m wide, and 40 m high were selected for this room, which will house the following equipment:

-Hydraulic turbine: This will transform the water energy into rotary mechanics, which in turn will later be transformed into electrical energy by means of an alternator coupled to its axis. According to the net hydraulic head and the water flow, the most suitable type of turbine can be estimated; Pelton and Francis are the most competitive turbines. For this plant in particular, the use of a reversible Francis turbine capable of working as a turbine and as a pump was selected. When it works as a turbine, it will be coupled to a generator, and when it works as a pump, it will be coupled to a motor that drives it to send water from the lower reservoir to the upper one. In this case, a synchronous generator coupled on the same axis as the turbine was chosen.

-Medium-voltage cells: These will be responsible for ensuring correct synchronization and the maximum operational reliability of the system. Depending on the type (protection, measurement, line, etc.), they will be installed electrically at different points in the powerhouse. Their main function will be to synchronize the frequency, voltage, and phase values within the network. Hydroelectric plants have very special demands on the generator cell compared to other types of power plants, especially in terms of switching operations. A circuit breaker capable of immediately interrupting the flow of electrical energy in the event of a short circuit in the generator or in the transformer will be integrated, avoiding secondary failures that may affect the complete operation of the plant [36].

-Surge tank: Its main functions will be to protect the turbine by absorbing pressure surges and to provide extra water during a short pressure drop, mainly in the pumping phase. In this case, a shaft with a diameter of 4 m and a height of 60 m located at the entrance of the powerhouse will be built.

### 5.1. UPHS Performance Analysis

According to Equation (1), the electrical power installed in reversible hydroelectric plants is directly proportional to the net hydraulic head (m) and the water flow (m^3^ s^−1^), which depends on the capacity of the reservoirs and the turbine cycle time at full load. To carry out a correct sizing of the plant, different scenarios were considered, by varying the previously mentioned parameters [37]. The energy production and consumption were analyzed for different hydraulic heads, depending on the useful volume of the lower tank. It was concluded that a hydraulic head lower than 300 m should not be considered, since the amount of energy stored is significantly reduced and a return on the investment is not guaranteed. A hydraulic head of above 600 m was not taken into account, since it is essential to reserve a volume of voids below the turbine to be able to keep pumping water and, in case of emergency, to be able to empty the lower tank. Considering the dimensions of the existing galleries of the Lieres mine, a useful volume of 250,000 m^3^ was selected for the lower reservoir (the same as the upper one). Table 1 shows the electrical energy production obtained by each turbine cycle (considering efficiencies of 90%, 98%, and 98.5% for the turbine, the transformer, and the alternator, respectively), the annual energy production (considering an operating period of 330 days per year, leaving 35 days for maintenance), and the energy consumption (considering a plant operation efficiency of 77%). The classic arbitrage pricing system between peak and valley hours has currently very low margins in the daily market. Given the high energy consumption, and in order to increase the profitability, it is proposed that the UPHS could participate in the ancillary services, and specifically in the secondary regulation band.

The head loss is associated with the diameter of the pipes, so a suitable one must be selected depending on the cycle time; additionally, the size of the reservoirs limits the water flow. The cycle time is the number of hours set for a 100% full load turbine operation (although the turbine could also operate at a lower load, increasing the number of hours per cycle, but this will not be considered here). In this case, the obtained water flow will be 9.9, 8.7, and 7.7 m^3^·s^−1^ for a cycle time of 7, 8, and 9 hours, respectively. The final selection will depend mainly on the strategy that the installation will adopt in the market. For example, if the plant is designed to participate in demand peaks, a higher nominal power will be required, resulting in a shorter cycle time and, therefore, a higher flow rate. Yet, if it is designed merely to offer availability to the electrical system, a higher cycle time would be chosen, resulting in a lower installed power. In this case, given that the UPHS plant would participate in the ancillary services, a turbine cycle time of 8 h was selected. Even if there is a significant reduction in the nominal power of the turbine compared to other scenarios with shorter cycle times, the availability of the plant for the electrical system will be considerably increased.

**Figure 5:**
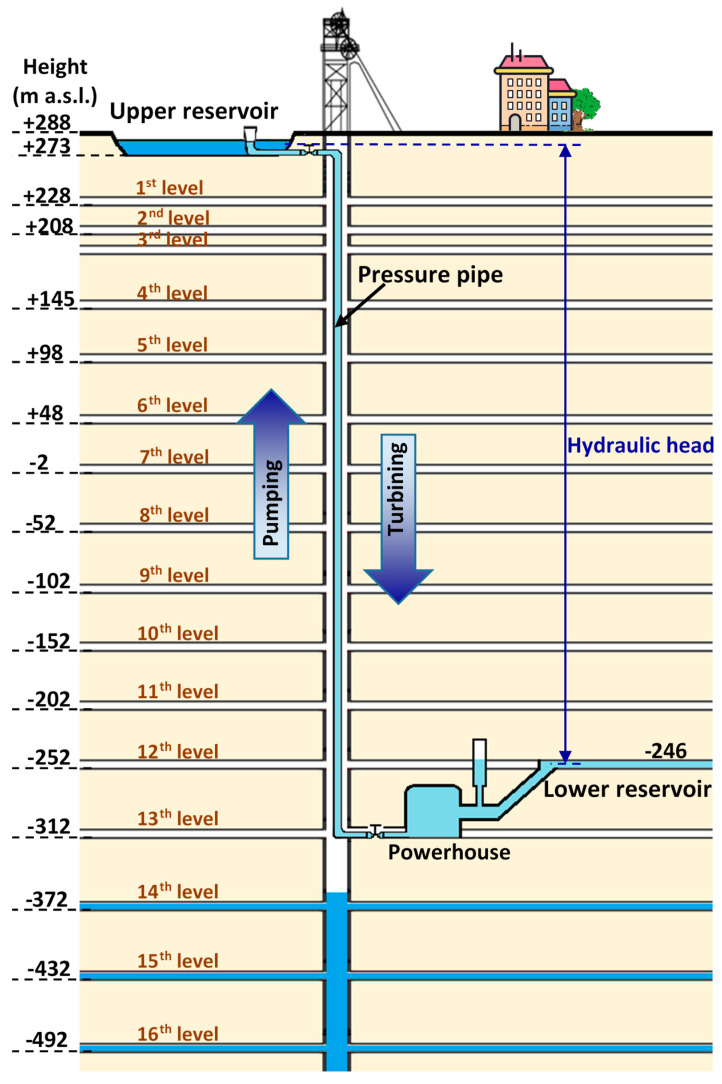
Schematic design of the UPHS plant inside the mine.

The net hydraulic head of the UPHS plant was calculated from the difference between the average gross jump of the installation and the load loss. The maximum gross head is given by the difference in height between the maximum level of exploitation of the upper reservoir and the minimum level of exploitation of the lower reservoir. The distance between the minimum level of exploitation of the upper reservoir and the highest level in the lower reservoir is known as the installation’s minimum gross jump. In this work, the lower reservoir will be built at the 12th level of the mine which is 540 m deep, and the powerhouse will be located at the 13th level, below the beforementioned reservoir, at a depth of 600 m (Figure 5).

To calculate the diameter of the pressure pipe (1.98 m), a typical energy balance problem related to the friction in the pipes was solved through an iterative process. Then, the water speed through the pressure pipeline was calculated at 2.82 m s^−1^. With these values and using the Darcy-Weissbach equations and the general equation for localized head loss, it was possible to find the total head loss of the installation (10.4 m), considering the intake work, the gratings, the valves, and the pressure pipe (friction, elbows, and kinetic energy of the water). Finally, the net hydraulic head of the facility (519.5 m) as well as the length of the pressure pipe (760.3 m) were calculated.

### 5.2. Production Parameters and Capital Expediture (CAPEX) of the UPHS Plant

The main production parameters necessary for the sizing of the Lieres’ UPHS plant, as well as the investment costs involved are shown in Table 2. As it was explained in Section 2, and according to Equation (1), the energy produced and consumed by the UPHS plant depends on the lower reservoir capacity, the net hydraulic head, and the water flow, which is estimated at 8.68 m^3^ s^−1^, considering a cycle time of 8 h. Taking a turbine efficiency of 90%, the obtained net power is 39.8 MW, so the energy production is 318.5 MWh day^−1^. Taking into account the efficiency of the alternator and the transformer, the energy sales per day and per year (the plant is operative 90% of the time), the annual net energy production is estimated to be 101.4 GWh. The UPHS plant efficiency was assumed to be 77%, so the energy consumption is 131.7 GWh.

To estimate the total investment costs of the UPHS plant, data provided by companies were used. For the civil works, average prices were set per unit built according to the type of excavation: i.e., EUR 5000 m^−1^ for the access tunnel, EUR 800 m^−1^ for the wells with a diameter of 1 m and EUR 15 m^−3^ excavated for the upper reservoir. The final cost includes the expenses due to waterproofing and the intake work.

## 6. Design of the Wind Farm

Wind energy is experiencing significant growth. At the end of 2019, the total wind power installed throughout the world was 651 GW, which means a 10% rise compared to 2018 [38]. It is the most mature and widely deployed “modern” renewable energy source worldwide that will undoubtedly continue to develop and become a key part of the world’s energy system in the future. In this work, a wind power generation facility was designed near the Lieres mine. The addition of a new wind farm to the UPHS will allow the system to be more flexible and will make it possible to purchase energy at a lower cost.

The first step in the process of selecting the most suitable location for the construction of a wind farm is the preliminary assessment of the available wind resource. In advanced projects, by means of the installation of measurement towers, a large volume of wind speed data can be available, so it is possible to estimate the revenue from electricity production that would be achieved in the wind farm, allowing a preliminary economic feasibility study to be conducted. In this case, the averaged values from the available regional wind maps were used. The World Wind Atlas of the International Energy Agency [39] was used due to its resolution. The ungrazed ridge line of an elevated area located at 10 km from the Lieres mine was selected. The area extends for 0.8 km^2^, with an altitude ranging from 1100 to 1250 m a.s.l. and it reaches an average wind speed of 8 m s^−1^ and an average power density of 1000 W m^−2^ at a height of 50 m.

This wind farm would be considered as a self-consumption facility, since it is intended to supply energy to the UPHS plant to pump water from the lower to the upper reservoir. According to this classification, the wind farm will not be affected by legal restrictions in the selected area as long as the total installation in the zone where it is located does not exceed 150 MW (and other existing wind farms installed in this zone do not exceed 10 MW).

For the optimal selection of the wind turbine, the quality and characteristics of the wind resource at the chosen site, as well as the specific use of the wind farm during the operation phase, should be taken into account. In the selected mountainous area, wind speeds can be highly variable, both in speed and direction. For this reason, vertical axis wind turbines (VAWTs) were chosen over the more popular horizontal axis models (HAWTs). This type of turbine constitutes a little-known innovative form of wind power generation with great potential, the main advantages of which are: (i) It is not affected by changes in the wind direction due to its symmetrical shape with respect to any vertical plane that crosses its axis and therefore does not require an orientation mechanism, which means significant savings. (ii) It can start generating energy from lower wind speeds. (iii) It requires simpler and cheaper maintenance, as the power train can be placed at ground level. (iv) It allows a faster recovery of wind speed after the wake of the wind turbine, so the turbines can be placed in close proximity to optimize the area available for the wind farm. Notwithstanding, there are disadvantages, including the fact that most VAWTs have self-starting difficulties and require an initial impulse to start rotating and generating electricity; however, when connected to the UPHS plant, they could take advantage of the power generated in the UPHS plant to give the generator-engine a boost. Likewise, they often have lower power coefficients (wind energy conversion efficiencies) than HAWTS [40,41].

### 6.1. Wind Farm Performance Analysis

The selected wind turbine was the ANew-B1 1.5MW VAWT designed in Krakow (Poland) [42]. This prototype is specially designed to be able to produce energy in a wide range of wind speeds (between 2.3 and 25 m s^−1^) and, contrary to the general trend of VAWTs, it has high energy conversion efficiencies (see Section 6.2.). Among other advantages, it has low rotational speeds (16 rpm at rated speed), so noise, collisions with birds, and interference with radio waves and radar, are reduced. Its rated power is 1.5 MW at 13.5 m s^−1^, although at speeds above 15 m s^−1^ it has a power output of around 1.8 MW. The hub height is 50 m and the rotor has a diameter of 52 m. Its lifespan is 20 years, whereas the UPHS plant’s lifespan is 40 years, so a total replacement of the wind turbines will be needed after 20 years of operation. This means an increase in the initial investment for the wind farm by 50% (it is not necessary to replace wiring, substations, foundations, etc.).

The layout of the wind turbines will be staggered to optimize the available land area. Following the manufacturer’s recommendations and introducing a recovery safety factor, a separation of four times the rotor diameter between the VAWTS (208 m) will be established. Therefore, 40 wind turbines can be allocated giving a total installed power of 60 MW.

### 6.2. Production Parameters and CAPEX of the Wind Farm

Table 2 shows the production parameters and the investment costs of the wind farm. A rotor swept area of 1700 m^2^, the aforementioned power density, an average energy conversion efficiency of 57%, and the fact that the wind speed at the site is higher than the VAWT connection speed (3.2 m s^−1^) 60% of the time were considered. Then, the average power generated by each wind turbine will be 581.4 kW. Considering a utilization factor of 90% (the farm is operational 330 of the 365 days of the year) the average energy generated by each wind turbine will be 4.61 GWh year^−1^ (184.2 GWh year^−1^ generated by the 40 turbines). Since the total installed power is 60 MW, the net equivalent hours of the wind farm will be 3070 h year^−1^.

The investment costs of the wind farm were calculated considering the average prices for vertical axis turbines and for the infrastructure of the installation provided by wind engineering companies (ISASTUR, TSK, VESTAS).

## 7. Hybrid System

The idea of combining renewable energy resources in the form of a hybrid system, which constitutes a reliable and cost-effective power generation system, is not new. Previous studies have proposed a hybrid system combining photovoltaic and PHS [43]. The combined system presented here is composed of an UPHS and wind and allows a reduction in operation costs. The engineering and commissioning costs of the hybrid system are included in Table 2.

To keep automatized control of both the hydro and wind generation plants, it will be necessary to install a power plant controller (PPC) that manages the downstream production and the entire system. This task will be carried out by a software that intelligently interconnects the different energy sources, manages the input and output production of each plant coordinately, and controls the possible constraints of the grid. Thus, the generation plants can digitally balance the different demands of the electricity sector.

The hybrid system has a series of connections that allow its operation to be optimized. Given its complexity, a simplified electrical installation was elaborated. The 40 wind turbines will be clustered into five groups of eight turbines in order to reduce the costs of the connection lines. Each wind turbine will generate electricity at a voltage of 400 V, which will be converted to medium voltage (30 kV) by means of a transformer. From each wind turbine there is a 1 × 400 mm^2^ aluminum line, which will be connected to the rest of the lines of the same group of wind turbines in a cell (one for each group). There will also be three auxiliary cubicles: one to control the frequency–power deviations of the line, one to house an auxiliary services transformer and, finally, one to protect the substation’s main transformer. Finally, there will be a single line that will extend to the 70 MVA 30/132 kV elevator substation, which will be responsible for elevating the power generated to high voltage, thus minimizing the losses due to the Joule effect during transport.

The power grid will then be subdivided into two parts: the line that transports the surplus wind energy directly to the point of discharge to the external grid (12 km), and the line that feeds the UPHS plant (10 km). In nominal conditions, they will transport 8 and 52 MW, respectively. Both conductors were dimensioned, obtaining an aluminum–steel LA-280 type. At the end of the supply line to the pumping station, a 75 MVA 132/13.8 kV step-down substation will be installed, which will directly supply power to the pump. This transformer will also function as a booster substation during the generation process. Finally, a line with the same characteristics will connect this installation to the grid access point. Two different access points (one for each line) at the same substation were chosen for the energy discharge. The minimum distance to access the regional electricity network is 2 km.

The total investment cost for the construction of the high-voltage lines, the grid access, the PPC, and all the substations aforementioned is estimated to be EUR 12.6 M.

## 8. Economic Feasibility

The income of the installation was calculated from the value obtained directly through energy sales, and by the remuneration established for this type of power plant following the Spanish Law (RD 413/2014), which regulates the electricity production from renewable energy sources. This value was estimated assuming that the system will participate in the daily electricity market and in the ancillary services of the Spanish electricity system. Given that the hydroelectric power plant will have an installed capacity of less than 50 MW, it will receive a remuneration of EUR 116,900 MW^−1^ year^−1^ for a maximum period of 25 years. Likewise, the wind farm would be entitled to a remuneration of EUR 98,638 MW^−1^ year^−1^ for a period of 20 years.

Regarding the operation and maintenance (O&M) costs for the entire hybrid system, which are detailed in Table 3, values provided by manufacturers and other companies within the energy sector already mentioned have been taken into account. To reduce O&M costs, it is proposed to share workers between both facilities. The annual operational expenditures (OPEX) estimated for the UPHS, and the wind farm are EUR 0.51 and 1.59 M, respectively, reaching a total combined cost of EUR 2.15 M year^−1^ (including the evacuation system).

In the economic profitability analysis of the hybrid system, the following parameters were considered: a price of EUR 50 and 75 MWh^−1^ for energy sales from wind surplus and from the UPHS plant, respectively [22], 25% on taxes, an interest rate on the loan of 3% and a credit period of 15 years.

The economic results are shown in Table 4. The total revenues reach EUR 20,802 k year^−1^, which were estimated considering the participation in the day-ahead and ancillary services markets. Additional retributions were considered according to the regulation of the Iberian electricity system. Regarding the expenses, it should be noted that the network access tariffs, and the hydraulic canon were calculated following the legislation of the Iberian electricity system. The network access tariffs for the electricity produced were calculated as EUR 0.5 MWh^−1^ and the hydraulic canon as 2.2% of the total revenues. Finally, a cost of EUR 100 k per year was estimated for insurances.

The economic study for the UPHS only results in a negative profitability of EUR −1.8 M, but it is positive when this plant is combined with the wind farm. For the hybrid system, the obtained payback period will be 19 years and the net profit at the end of the lifespan of the hybrid system (40 years) will be EUR 53.7 M. When an investment of this magnitude is undertaken, possible legislative financial aids (Spanish, European) are always sought in order to ensure, or as in this case, improve the profitability. Although it is true that on many occasions they are not received, it is realistic to consider such a scenario due to the second life that abandoned coal mines acquire and the low-carbon emission power technology that is implemented. In this respect, a reduction in the emission of 29,000 equivalent tons of CO_2_ per year will be achieved, considering that a total of 153.8 GWh will be fed into the grid and the average emission factor of the Iberian electricity market of 0.19 tons CO_2_-e MWh^−1^ [44]. In this context, considering a 50% subsidy, a profit of EUR 97 M would be reached.

### Sensitivity Analysis

**Figure 6:**
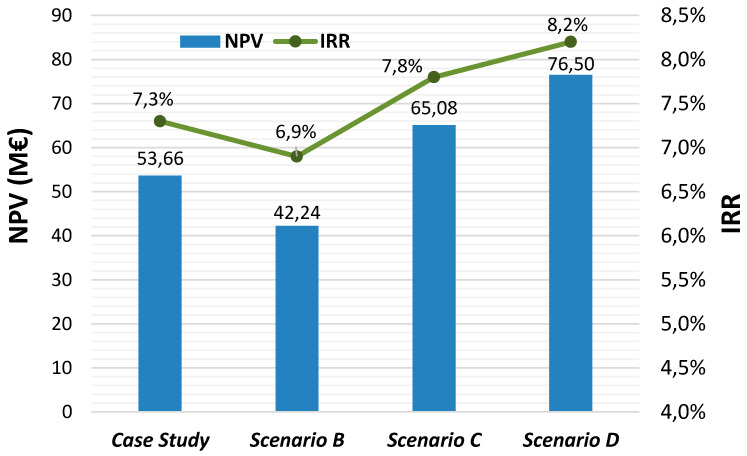
Sensibility analysis of the NPV and IRR parameters depending on the energy sales prices.

The net present value (NPV) and the internal rate of return (IRR) were obtained for different scenarios depending on the electricity prices in the day-ahead and ancillary services markets. For each scenario, different energy prices were considered, both for the UPHS plant and for the wind farm, and the calculated revenues of the hybrid system were calculated (Table 5). Likewise, the NPV and the IRR were recalculated keeping the rest of the variables constant (Figure 6). It is observed that profitability increases when the price of energy also increases. This effect is more notable in the profitability of the UPHS plant, since it is the one that supplies the most energy. The wind farm revenues are not so affected by market conditions since wind energy will only be used when the UPHS plant operates in consumption mode for pumping.

## 9. Conclusions

Mine water can be used as a source of renewable energy, such as in underground pumped hydroelectric energy storage systems (UPHS). The valuable use of this resource is particularly interesting in those mines that have to maintain a persistent pumping scheme. Despite the current non-existence of commercial-scale UPHS plants, these types of facilities represent a potential solution to the problems derived from the intermittent nature of some renewable energy sources such as solar or wind. This would enable the electrical system as well to further integrate renewable energies, which could result in an attractive idea for potential investors. The possibility of reusing abandoned mines is an extra incentive for companies and regional and state administrations, who may come to see in them the solution to the problems derived from the dismantling of underground mines not only in terms of job creation, but also in terms of rebuilding the socio-labor structure in the affected regions.

In particular, the UPHS presented here, with a net hydraulic head of 519.5 m and a total installed capacity of 39.8 MW, combined with 40 type A-NEW B1 wind turbines, will result in an NPV of almost EUR 54 M from an original amount of EUR 55.5 M. It should be noted that this system is limited by the existence of a number of environmental, geographical, social, and political constraints that define, in the first instance, the viability of its execution. These constraints are: (i) The existence of an underground infrastructure of unused tunnels and cavities with enough stability and capacity to house the lower reservoir and the other auxiliary equipment of the UPHS plant. (ii) The availability of a high-quality wind resource in a location close to the underground hydroelectric power station that makes its use viable to become a safe and stable source of income. (iii) The economic and socio-political influence exerted by the stakeholders, whose predisposition may be decisive regarding the approval and authorization for the execution of a system of such magnitude.

Despite having recognized and studied in some depth the functioning mechanisms of the Spanish electricity system, it was decided to leave out of the scope of this study those particular considerations that relate to fluctuations in electricity demand. The storage of renewable energy is the future, and cooperation between public and private actors will be the key to rebuilding economies for a cleaner and more sustainable tomorrow. The work revealed here is part of this line and presents a technically and economically viable option, which can be extrapolated to other regions.

## Figures and Tables

**Table 1:** Energy production and consumption as a function of the UPHS hydraulic head (lower reservoir capacity: 0.25 Mm^3^).

Net Hydraulic Head (m)	Energy Production (MWh/Cycle)	Energy Production (GWh/Year)	Energy Consumption (GWh/Year)
300	177.6	58.6	76.1
400	236.7	78.1	101.5
500	195.9	97.6	126.8
600	355.1	117.2	152.2

**Table 2:** Production parameters and the investment costs of the hybrid system.

Production Parameters			Investment Costs (EUR (k))	
UPHS Plant	Turbine cycle time (h)	8	New access	22,200
	Flow rate (m^3^ s^−1^)	8.68	Upper reservoir	4400
	Pressure pipe speed (m s^−1^)	2.82	Pressure pipe	1820
	Max. Gross hydraulic head (mH_2_O)	540.4	Immersion gallery	1860
	Min. Gross hydraulic head (mH_2_O)	519.4	Transformer station	590
	Aver. Gross hydraulic head (mH_2_O)	529.9	Powerhouse	2260
	Head losses (mH_2_O)	10.43	Surge chamber	450
	Net hydraulic head (mH_2_O)	519.5	Lower reservoir	22,200
	Gross turbine power (MW)	44.23	Ventilation ducts	245
	Turbine efficiency (%)	90	Turbine-pump	6750
	Net power (MW)	39.81	Motor-alternator	6360
	Energy production (MWh day^−1^)	318.5	Electrical substation	880
	Transformer efficiency (%)	98	Electrical system	2280
	Alternator efficiency (%)	98.5	Hydraulic system	440
	Energy sales (MWh day^−1^)	307.4	Automation and control	391
	Energy sales (GWh year^−1^)	101.4	Energy evacuation	391
	Pump mode power (MW)	51.7	UPHS plant total cost	73,517
	Energy consumption (GWh year^−1^)	131.7		
Wind Farm	Power density (W m^−2^)	1000	VAWT unit cost per MW	1014
	Swept area (m^2^)	1700	-Turbine	
	Energy conversion factor	57	-Transformer station	
	Time with wind speed > 3.2 m s^−1^ (%)	60	-Blades	
	Period of operation (days year^−1^)	330	-Control system	
	Utilization factor	0.9	VAWTs total cost	60,840
	Gross wind turbine power (kW)	1500	Cost of civil works per MW	167
	Average wind turbine power (kW)	581	Total cost of civil works	10,020
	Wind turbine energy production (MWh year^−1^)	4605	Wind farm collector system	1280
	Gross wind farm power (MW)	60.0	-Medium voltage line (30 kV)	
	Average wind farm power (MW)	23.25	-Wires (30,000 m)	
	Wind farm energy production (GWh year^−1^)	184.2	-Auxiliary servicer transformer	
	Equivalent hours (h year^−1^)	3070	-Low voltage access point	
	Energy dumped into UPHS (GWh year^−1^)	131.7	-8 cells high voltage line	
	Energy fed to the grid (GWh year^−1^)	52.4	Balance of plant (BOP)	11,300
			Wind farm total cost	72,140
Hybrid system			Engineering	1500
			Commissioning	650
			Hybrid system total cost	147,807

**Table 3:** Operation and maintenance (O&M) costs of the hybrid system.

O&M Costs (EUR(k)/Year)	
Wind Turbines	1520
Spare parts	60
Civil works	75
Evacuation system	50
Staff	300
External services and general expenses	150
Total O&M costs	2155

**Table 4:** Economic results of the hybrid system.

Economic Results (EUR(k))	
Revenues	20,802
Energy sales (day-ahead market and ancillary services)	10,230
Retribution Hybrid System (additional retribution)	10,572
Expenses	15,630
Operation and caintenence costs	2155
Network access tariffs	77
Hydraulic canon	458
Insurance	100
Amortization	12,841
Earnings Before Interests and Taxes (EBIT)	5172
Financial costs	1623
Earnings Before Taxes (EBT)	3549
Taxes	887
Economic result	2661
Cash flow	15,502

**Table 5:** Sensitivity analysis. Total annual energy sales revenues for different scenarios.

	Case Study	Scenario B	Scenario C	Scenario D
UPHS (EUR/MWh)	75	70	80	85
Wind Power (EUR/MWh)	50	45	55	60
**Revenues**				
UPHS energy sales (MWh/year)	101,449	101,449	101,449	101,449
Wind Power energy sales (MWh/year)	52,435	52,435	52,435	52,435
Hybrid system energy sales (MWh/year)	153,884	153,884	153,884	153,884
UPHS energy sales (EUR(k))	7609	7101	8116	8623
Wind Power energy sales (EUR (k))	2622	2360	2884	3146
Hybrid system energy sales (EUR (k))	10,230	9461	11,000	11,769
UPHS retribution (EUR (k))	4654	4654	4654	4654
Wind Power retribution (EUR (k))	5918	5918	5918	5918
Hybrid System retribution sales (EUR (k))	10,572	10,572	10,572	10,572
TOTAL REVENUES (EUR (k) /year)	20,802	20,033	21,572	22,341

## References

[1] FCH Commercialisation of Energy Storage in Europe. https://www.fch.europa.eu/sites/default/files/CommercializationofEnergyStorageFinal_3.pdf.

[2] BloombergNEF New Energy Outlook 20209. https://about.bnef.com/new-energy-outlook.

[3] Matos C.R., Carneiro J.F., Silva P.P. (2019). Overview of Large-Scale Underground Energy Storage Technologies for Integration of Renewable Energies and Criteria for Reservoir Identification. J. Energy Storage.

[4] Portero U., Velázquez S., Carta J.A. (2015). Sizing of a wind-hydro system using a reversible hydraulic facility with seawater. A case study in the Canary Islands. Energy Convers. Manag..

[5] Pieper C., Rubel H. Electricity Storage: Making Large-Scale Adoption of Wind and Solar Energies a Reality. https://image-src.bcg.com/Images/BCG_Electricity_Storage_Mar_2010_tcm9-236613.pdf.

[6] Newman J., D’Aprile P. *The New Economics of Energy Storage*. McKinsey & Company. https://www.mckinsey.com/business-functions/sustainability/our-insights/the-new-economics-of-energy-storage.

[7] Fessenden R.A. (1910). The commercial solution of the problem of utilising, for the production of power, the energy of solar radiation, the wind and other intermittent natural sources. Electrician.

[8] Deane J.P., Gallachóir B.P.Ó., McKeogh E.J. (2010). Techno-economic review of existing and new pumped hydro energy storage plant. Renew. Sustain. Energy Rev..

[9] Barbour E., Wilson I.G., Radcliffe J., Ding Y., Li Y. (2016). A review of pumped hydro energy storage development in significant international electricity markets. Renew. Sustain. Energy Rev..

[10] Kadiyala A., Kommalapati R., Husque Z. (2016). Evaluation of the Life Cycle Greenhouse Gas Emissions from Hydroelectricity Generation Systems. Sustainability.

[11] Meyer F. (2013). Storing Wind Energy Underground.

[12] Rohit A.K., Devi K.P., Rangnekar S. (2017). An overview of energy storage and its importance in Indian renewable energy sector: Part I—Technologies and Comparison. J. Energy Storage.

[13] Menéndez J., Ordóñez A., Álvarez R., Loredo J. (2019). Energy from closed mines: Underground energy storage and geothermal applications. Renew. Sustain. Energy Rev..

[14] Uddin N. (2003). Preliminary design of an underground reservoir for pumped storage. Geotech. Geol. Eng..

[15] Pujades E., Willems T., Bodeux S., Orban P., Dassargues A. (2016). Underground pumped storage hydroelectricity using abandoned works (deep mines or open pits) and the impact on groundwater flow. Hydrogeol. J..

[16] Bodeux S., Pujades E., Orban P., Brouyère S., Dassargues A. (2017). Interactions between groundwater and the cavity of an old slate mine used as lower reservoir of an UPSH (Underground Pumped Storage Hydroelectricity): A modelling approach. Eng. Geol..

[17] Pujades E., Orban P., Bodeux S., Archambeau P., Erpicum S., Dassargues A. (2017). Underground pumped storage hydropower plants using open pit mines: How do groundwater exchanges influence the efficiency?. Appl. Energy.

[18] Harza R.D. (1960). Hydro and pumped storage for peaking. Power Eng..

[19] Williams D. *Coal Mine to Be Transformed into 200 MW Pumped Hydro Plant*. Power Engineering International. https://www.powerengineeringint.com/coal-fired/coal-mine-to-be-transformed-into-200-mw-pumped-hydro-plant.

[20] Winde F., Kaiser F., Erasmus E. (2016). Exploring the use of deep level gold mines in South Africa for underground pumped hydroelectric energy storage schemes. Renew. Sustain. Energy Rev..

[21] Braat K.B., Van Lohuizne H.P.S., De Haan J.F. (1985). Underground Pumped Hydro-storage Project for the Netherlands. Tunn. Tunn..

[22] Menéndez J., Fernández-Oro J.M., Loredo J. (2020). Economic Feasibility of Underground Pumped Storage Hydropower Plants Providing Ancillary Services. Appl. Sci..

[23] Menéndez J., Fernández-Oro J.M., Galdo M., Loredo J. (2020). Efficiency analysis of underground pumped storage hydropower plants. J. Energy Storage.

[24] Özarslan A., Aydiner H.Y., Köken E., Alber M., Kwasniewski M., Lydzba D. (2013). Stability analysis of deep coal mine main roadways for pumped hydropower lower reservoir storage. Rock Mechanics for Resources, Energy and Environment, Proceedings of EUROCK2013.

[25] Gandy C.J., Younger P.L. (2007). Predicting Groundwater Rebound in the South Yorkshire Coalfield, UK. Mine Water Environ..

[26] Ordóñez A., Jardón S., Álvarez R., Andrés C., Pendás F. (2012). Hydrogeological definition and applicability of abandoned coal mines as water reservoirs. J. Environ. Monit..

[27] Álvarez R., Ordóñez A., De Miguel E., Loredo C. (2016). Prediction of the flooding of a mining reservoir in NW Spain. J. Environ. Manag..

[28] Motaung S., Maree J., De Beer M., Bologo L., Theron D., Baloyi J. (2008). Recovery of Drinking Water and By-products from Gold Mine Effluents. Int. J. Water Resour. Dev..

[29] Younger P.L., Banwart S.A., Hedin R.S. (2002). Mine Water, Hydrology, Pollution, Remediation.

[30] Jardón S., Ordóñez A., Álvarez R., Cienfuegos P., Loredo J. (2013). Mine Water for Energy and Water Supply in the Central Coal Basin of Asturias (Spain). Mine Water Environ..

[31] Pérez-Estaún A., Bastida F., Alonso J.L., Marquínez J., Aller J., Álvarez-Marrón J., Marcos A., Pulgar J.A. (1988). A thin-skinned tectonics model for an arcuate fold and thrust belt: The Cantabrian Zone (Variscan Ibero–Armorican Arc). Tectonics.

[32] Colmenero J.R., Suárez-Ruiz I., Fernández-Suárez J., Barba P., Llorens T. (2008). Genesis and rank distribution of Upper Carboniferous coal basins in the Cantabrian Mountains, Northern Spain. Int. J. Coal Geol..

[33] Jinyang F., Heping X., Jie C., Deyi J., Cunbao L., Tiedeu W., Ambre J. (2020). Preliminary feasibility analysis of a hybrid pumped-hydro energy storage system using abandoned coal mine goafs. Appl. Energy.

[34] Pujades E., Orban P., Jurado A., Ayora C., Brouyere S., Dassargues A. (2017). Water chemical evolution in Underground Pumped Storage Hydropower plants and induced consequences. Energy Procedia.

[35] Niemann A. *Underground Pumped Hydroelectric Storage Using Existing Coal Mining Infrastructure of Prosper Haniel Mine, Germany*. University of Duisburg-Essen. 4th Meeting Coal Regions in Transition—Energy Storage. https://ec.europa.eu/energy/sites/ener/files/documents/6.2_niemann_energy_storage.pdf.

[36] Pei (Power Engineering International) Switchgear Solution for Hydropower Plants. https://www.powerengineeringint.com/world-regions/asia/switchgear-solution-for-hydropower-plants.

[37] Madlener R., Spetch J.M. (2020). An Exploratory Economic Analysis of Underground Pumped-Storage Hydro Power Plants in Abandoned Coal Mines. Energies.

[38] Global Wind Energy Council (GWEC) Global Wind Report 2019. https://gwec.net/global-wind-report-2019.

[39] International Renewable Energy Agency (IRENA) Global Atlas for Renewable Energy. DTU Global Wind Atlas 1 km Resolution..

[40] Beurskens J., Brand A., Crawley G.M. (2015). Wind energy. The World Scientific Handbook of Energy.

[41] Möllerström E., Gipe P., Beurskens J., Ottermo F. (2019). A historical review of vertical axis wind turbines rated 100 kW and above. Renew. Sustain. Energy Rev..

[42] ANew Institute Vertical Axis Wind Turbines. https://www.anew-institute.com.

[43] Makhdoomi S., Askarzadeh A. (2020). Daily performance optimization of a grid-connected hybrid system composed of photovoltaic and pumped hydro storage (PV/PHS). Renew. Energy.

[44] Red Eléctrica de España Emisiones y Factor de Emisión de CO2 eq. de la Generación (Emissions and CO2-e Emission Factor of Generation). Spanish National Electricity System..

